# Introduction of safety and quality standards for private health care providers: a case-study from the Republic of Srpska, Bosnia and Herzegovina

**DOI:** 10.1186/s12939-018-0806-0

**Published:** 2018-10-05

**Authors:** Severin Rakic, Budimka Novakovic, Sinisa Stevic, Jelena Niskanovic

**Affiliations:** 1 Public Health Institute of the Republic of Srpska, Jovana Ducica 1, 78000, Banjaluka, Bosnia and Herzegovina; 2 Medical Faculty, University of Novi Sad, Novi Sad, Republic of Serbia; 3 Agency for Certification, Accreditation and Healthcare Quality Improvement in the Republic of Srpska, Banjaluka, Bosnia and Herzegovina

**Keywords:** Quality of health care, Patient safety, Certification, Private health care providers, Standards, Diffusion of innovation, Mixed methods, Multiple case study, Regulation, Enforcement

## Abstract

**Background:**

Regulation of private health care providers (PHPs) in middle-income countries can be challenging. Mandatory safety and quality standards for PHPs have been in place in the Republic of Srpska since 2012, but not all PHPs have adopted them yet. Adoption rates have differed among different types of providers. We studied three predominant types of PHPs to determine why the rate of adoption of the standards varies among them.

**Methods:**

This study used a mixed methods approach, which allowed the integration of both quantitative and qualitative data, to develop an explanatory case study. The case study covered three types of private PHPs: pharmacies, dental practices and specialist practices. Primary data were collected through face-to-face semi-structured in-depth interviews and a self-administered postal survey of private health care providers. Our study’s theoretical framework was based on the diffusion of innovation theory.

**Results:**

The rate of adoption of mandatory standards varied among different types of PHP mainly due to four factors: (1) level of concern about negative financial consequences, such as the risk of fines or of losing contracts with the Health Insurance Fund of the Republic of Srpska; (2) availability of information on the standards and implementation process; (3) level of the relevant professional association’s support for the introduction of standards; and (4) provider’s perceptions of the relevant health chamber’s attitude toward the standards. Opinions conveyed to PHPs by peers slightly negatively influenced adoption of the standards at the attitude-forming stage. Perceived gains in professional status did not have a major influence on the decision to adopt standards. All three types of PHPs perceived the same disadvantages of the introduction of safety and quality standards: associated expense, increased administrative burden and disruption of service provision.

**Conclusions:**

When introducing mandatory quality and safety standards for PHPs, national health authorities need to: ensure adequate availability of information on the relative advantages of adhering to standards; support the introduction of standards with relevant incentives and penalties; and work in partnership with relevant professional associations and health chambers to get their buy-in for regulation of quality and safety of health services.

**Electronic supplementary material:**

The online version of this article (10.1186/s12939-018-0806-0) contains supplementary material, which is available to authorized users.

## Background

Concerns about the safety and quality of health care services are as old as medicine [1]. Improving universal health coverage through the development of safety and quality standards was a component of the World Health Assembly’s 1997 global goal of “Health for All by the Year 2000” [2]. Accounting for the quality of health services remains a challenge when measuring progress in achieving universal health coverage [3]. Sustained efforts on the parts of health care workers, researchers and policy makers are required to make the improvements in the safety and quality of services that result in better health outcomes for users and improvement of health care systems [4].

The Republic of Srpska (RS), one of the constituent parts of Bosnia and Herzegovina, has its own legislative and executive functions and responsibilities, including those related to health care. Private for-profit health care providers in the RS substantially contribute to service delivery, particularly at the primary health care level. The majority of dental services for the adult population are provided by private dental practices. The network of private pharmacies assures access to medicines, as there are only a few public pharmacies. The number of private specialist practices (e.g. pediatricians, gynecologists, ophthalmologists, dermatologists) has also grown since the RS Health Insurance Fund started contracting these services in 2010, a policy that was introduced to assure access to the services in rural areas. These three types of PHPs—pharmacies, dental practices and specialist practices—account for over 95% of all PHPs in the RS.

There are three health chambers in the RS whose main role is licensing of health care professionals. Membership in their respective chamber is mandatory for all practicing pharmacists, dentists and medical doctors. There are also professional associations in the RS formed by pharmacists, dentists and medical doctors. These are civil society organizations, with voluntary membership and self-serving interests. Some professional associations only bring together PHPs (such as the Association of Private Medical Doctors of the RS), while others include both public and private health care providers (such as the Pharmaceutical Society of the RS).

The Ministry of Health and Social Welfare of the RS has been concerned with the quality and safety of health care services for more than two decades. These concerns were comprehensively articulated just over a decade ago in the *Policy for Improvement of Quality and Safety of Healthcare in the Republic of Srpska until 2010* [5]. One of the Policy’s objectives was to develop and adopt legislation to support the establishment and improvement of safety and quality systems in health care. The current RS *Law on Healthcare* [6], enacted in 2009, provides the desired innovative legal framework. The *Law on Healthcare* introduced both a mandatory certification based on a core set of basic standards for both public and private health care providers and a voluntary accreditation option based on broader and more demanding quality standards. None of the regulations on health care safety and quality in the RS specified clear deadlines for adoption of standards.

Accreditation of health care providers is a mechanism proven to enhance safety and quality [2]. It entails public recognition by a health care accreditation body that a health care organization has achieved pre-defined standards that reflect observance of measures to maintain safety and quality. Accreditation is secured through an independent external peer assessment of the organization’s level of performance in relation to the standards [2, 7, 8]. Introduction of safety and quality standards, together with external assessment of health care providers, leads to better health outcomes and realization of universal quality coverage and equity in health [7–10]. Facilities seeking accreditation are compelled to improve safety standards, regardless of whether they serve poor or vulnerable populations or are located in rural or distant areas. Thus, the potential inequality in standards of care across facilities is reduced and equity is potentially enhanced.

Health care professionals have different attitudes toward accreditation and the introduction of common safety and quality standards [11–16]. In public health systems, however, providers are obliged to participate. Private health care providers (PHPs) present other challenges to health care policy makers. Continuous engagement with providers, and a coordinated and integrated approach to improving the safety and quality of private health care services, represents a challenge [17]. One aspect of an integrated approach is the implementation of regulation; this can prove especially challenging in middle-income countries [18–20] with previously unregulated private health care sector, where regulation of the private providers is a more recent development.

The RS *Law on Healthcare* [6] created a system of certification intended to (1) improve the safety of patients and staff, (2) increase users’ trust in the health system, (3) enable continuous quality improvement of health care services, (4) work towards equal conditions for provision of health services throughout the RS, and (5) support environmental protection. Certification is carried out through an established procedure to evaluate and confirm that a health care provider fully meets the predefined and published standards [6, 21]. The Agency for Certification, Accreditation and Health Care Quality Improvement in the Republic of Srpska (ASKVA) assesses providers’ compliance with the certification standards. The ASKVA has trained external experts to conduct the assessment process. The assessment procedures and roles of the ASKVA and health care providers in the certification process have been precisely defined by a bylaw issued by the Ministry of Health and Social Welfare of the RS [22]. Health care organizations with a complex structure, such as public facilities offering a mix of different types of services, are allowed to apply to ASKVA for partial assessment of compliance with standards; this option to leave some parts out of the assessment has not been available to PHPs.

The ASKVA developed specific certification standards for different types of public providers (namely hospitals, primary health care centers, and the institute for dentistry) and for private health care providers (including pharmacies, specialist practices, family medicine practices, dental practices, and specialized dental practices). The certification standards have three areas of focus (Table 1): patient safety (including for instance, implementation of regulatory measures for control of nosocomial infections in the health facilities), staff safety (such as, implementation of occupational health and safety measures in the health facilities) and environmental protection (for example, adequate disposal of medical waste by the health facilities).

**Table 1:** Structure of certification standards for pharmacies, specialist practices and dental practices

	Pharmacies		Specialist practices		Dental practices
1.	Management of the pharmacy	1.	Management of the practice	1.	Management of the practice
	1.1 Legal status of the pharmacy		1.1 Legal status of the practice		1.1 Legal status of the practice
	1.2 Human resource management		1.2 Human resource management		1.2 Human resource management
	1.3 Control of documents		1.3 Control of documents		1.3 Control of documents
	1.4 Risk management (adverse events)		1.4 Risk management (adverse events)		1.4 Risk management (adverse events)
	1.5 Fire protection and occupational safety		1.5 Fire protection and occupational safety		1.5 Fire protection and occupational safety
			1.6 Safe environment for staff and patients		1.6 Safe environment for staff and patients
			1.7 Equipment in the practice		1.7 Equipment in the practice
2.	Services provided by the pharmacy	2.	Safety of services	2.	Dental services
					2.1 Safety of services
	2.1 Communication with patients		2.1 Information and communication with patients		2.2 Information and communication with patients
	2.2 Self-care and self-medication				
	2.3 Patients’ rights				
3.	Documentation in the pharmacy	3.	Medical documentation	3.	Medical documentation
4.	Staff of the pharmacy	4.	Staff of the practice	4.	Staff of the practice
5.	Physical conditions	5.	Control of healthcare-acquired infections	5.	Control of healthcare-acquired infections
	5.1 Premises of the pharmacy		5.1 Cleaning of premises and surfaces		5.1 Cleaning of premises and surfaces
	5.2 Physical accessibility				
			5.2 Hand hygiene		5.2 Hand hygiene
			5.3 Personal protective equipment		5.3 Personal protective equipment
			5.4 Prevention of exposure to blood borne viruses		5.4 Prevention of exposure to blood borne viruses
			5.5 Decontamination and sterilization of instruments and equipment		5.5 Decontamination and sterilization of instruments and equipment
6.	Safety of medicines and pharmaceutical waste management		5.6 Medical waste management		5.6 Medical waste management

Note: Based on ASKVA’s certification standards for pharmacies [50], specialist practices [51] and dental practices [52]

**Fig. 1:**
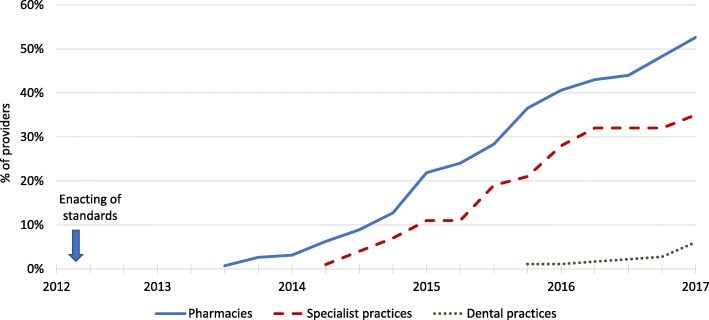
Private health care providers that adopted the certification standards by end of December 2016. Legend: Developed on the basis of ASKVA’s [23] and Ministry of Health and Social Welfare’s registry [33]

These mandatory safety and quality standards have been in place in the RS since 2012, but have not yet been adopted by all providers, as the non-adopters faced no real penalties. The new regulation required providers to change existing practices in order to comply with the standards. Public hospitals and primary health care centers were supported by the RS health authorities to comply with requirements of the new standards. The authorities provided technical assistance in the development of organizational structures and capacities for continuous quality improvement as well as the development of documentation required by the standards. Regardless of the assistance, by the end of 2016 only about 35% of public hospitals and 40% of public primary health care centers had completed mandatory certification [23]. Adoption rates among different types of the PHPs are shown in Fig. 1.

This study found that public and private health care providers held similar perceptions about the certification standards. The idea of legally mandated standards was new for all providers in the RS, although such practices exist in other countries.

A limited number of studies have previously examined how externally mandated innovations are adopted by health care providers [24–26]; a few others have observed government-imposed continuous quality improvement initiatives [27, 28]. We studied three types of PHPs (pharmacies, dental practices and specialist practices) to determine why the rate of adoption of mandated certification standards (considered an external innovation) varied among the PHPs.

### Theoretical framework

This research was based on Rogers’ diffusion of innovation theory [29], which defines an innovation as an idea, practice or object that is perceived as new by an individual or other unit of adoption. Diffusion is defined as a process by which an innovation is communicated through certain channels over time among the members of a social system. While other innovation adoption theories exist [30, 31], Rogers’ diffusion of innovation theory seemed most suitable for the research question, in which the main dependent variable was the rate(s) of adoption of an innovation in different social systems (Table 2). The aim of this type of diffusion research is to study why the same innovation was adopted more rapidly in certain systems than in others.

**Table 2:** Types of diffusion research

Type	Main dependent variable	Frequency in diffusion publications (%)
1	Earliness of knowing about an innovation by members of a social system	5
2	Rate of adoption of different innovations in a social system	1
3	Innovativeness of members of a social system	58%
4	Opinion leadership in diffusing innovations	3%
5	Diffusion networks	< 1%
6	Rate of adoption of innovations in different social systems	2%
7	Communication channel use	7%
8	Consequences of an innovation	< 1%
9	Others	22%
	Total	100%

Note: Based on analysis conducted by Rogers [29]

According to Rogers [29], there are four main elements of the diffusion process. The first is the innovation, which in this research is the introduction of mandatory safety and quality standards. The second is the communication channel involved, which in this case was defined as the means by which information on mandatory safety and quality standards reached the PHPs. The third factor is time, which this research defined as the time between when a provider first learns of the standards to their adoption or decision to reject. The final factor, according to Rogers, is the social system. In this research, the social system is the health system of the RS, including the interrelated public and private health care providers, as well as their professional associations and chambers.

Rogers posits that the innovation-decision process consists of five stages: knowledge, persuasion, decision, implementation and confirmation. The individual initially plays a relatively passive role when being exposed to knowledge about an innovation. In the persuasion stage the individual actively seeks out information about the new idea and develops a general perception of innovation on the basis of its perceived attributes, such as relative advantage, observability, compatibility, trialability and complexity. While mass media channels tend to be important at the knowledge stage, interpersonal channels become more important at the persuasion stage. At the decision stage, an innovation can be adopted or rejected by an individual independently (optional innovation-decision), by consensus among members of the system (collective innovation-decision), by a few individuals within a system who possess power, status or technical expertise (authority innovation-decision) or in a sequential combination of two or more tandem decisions (contingent innovation-decisions). Various communication channels within a social system are directly involved in all four types of innovation-decisions. Contingent innovation-decisions are the most complex, as the final choice to adopt or reject innovation can only be made after a prior decision or decisions.

This study compared the adoption of mandatory safety and quality standards in three different social sub-systems: dental practices, pharmacies and specialist practices. The three initial stages in the innovation-decision process (knowledge, persuasion and decision) were identified as leading to the PHPs’ choice to adopt the standards or not. Implementation of the standards, the fourth stage in the process, was not considered in this study. The rate of adoption was defined as the proportion of a particular type of PHPs that have adopted the standards as verified by the ASKVA.

One specific type of innovation is the preventive innovation, in which a new idea is adopted in order to lower the probability of some unwanted future event. Preventive innovations typically have a slow rate of adoption because individuals perceive their relative advantages to be distant in time. Adoption of certification standards can be regarded as a preventive innovation, because the PHPs adopted a new idea relatively slowly in order to avoid the possible occurrence of unwanted events in the future.

In this study, we hypothesized that the rate of adoption of mandatory safety and quality standards would be influenced by five elements:

(1) Perceived gains in professional status. In diffusion of innovation theory terminology, this represents observability. This attribute of the innovation influences the perception stage of the innovation-decision process.

(2) Fear of negative financial consequences. According to the diffusion of innovation theory, this is a relative advantage, another attribute of the innovation that influences the perception stage of innovation-decision process.

(3) Availability of information on safety and quality standards. This falls under the mass media and interpersonal communication channels component of diffusion of innovation. These are necessary for gaining knowledge on the innovation.

(4) Opinions conveyed to private health care providers by peers. In terms of the diffusion of innovation theory, these are interpersonal communication channels that influence the persuasion and decision stages.

(5) Perceived attitudes of the health chambers and professional associations. In diffusion of innovation theory these represent parts of a social system, with power to make one of the tandem decisions that comprise the contingent innovation-decision process.

Table 3 shows the eight variables recognized by the diffusion of innovation theory that were selected, based on the hypothesized influences, to frame the study.

**Table 3:** Adaptation of definitions from the diffusion of innovation theory to the study context

Property of innovation	Variables	Adapted definitions
Perceived attributes of innovation	Relative advantage	Degree to which adoption of mandatory safety and quality standards is perceived as better than retaining status quo. It is a ratio of the expected benefits (e.g. better professional reputation, better management, improved patient satisfaction, economic profitability) and the costs of adoption (e.g. disadvantages to the provider).
	Observability	Degree to which the results of adoption of mandatory safety and quality standards are visible to others (e.g. patients, peers, inspection, health insurance fund, line ministry)
Communication channels	Mass media channels	All the means of transmitting messages, involving a mass medium (television, radio, Internet or press), through which audience of many PHPs and public got information on certification process.
	Interpersonal channels	Face-to face exchange of information on mandatory safety and quality standards between owners of PHPs and other individuals (e.g. peers, both adopters and non-adopters of the standards; representatives of other organizations).
Innovation decision process	Knowledge	Exposure of PHP’s owner to information on safety and quality standards and gaining of understanding on how the certification process functions.
	Persuasion	Forming of PHP’s owner favorable, neutral or unfavorable attitude towards the certification standards and process.
	Decision	Engaging of PHP’s owner in activities leading to a choice to adopt or reject the certification standards and process.
Social system	Collective innovation-decision	Choice to adopt or reject certification process that is made by consensus among the members of a health chamber or members of a professional association of private health care providers.

## Methods

### Study design

**Fig. 2:**
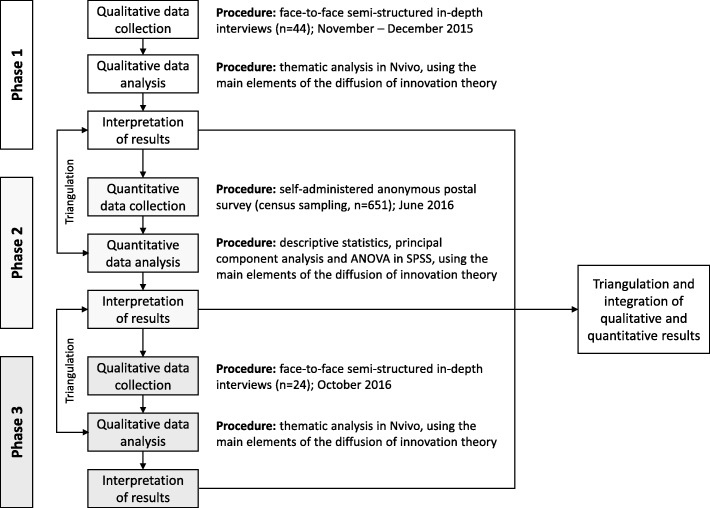
Overview of the study process

A sequential mixed methods design, illustrated in Fig. 2, was used in order to draw on the strengths of both qualitative and quantitative methods and develop a more complete understanding of the situation. The research began with the collection of qualitative data during exploratory interviews with 18 adopters and 26 non-adopters of the standards. The insight into the PHPs’ perspectives from Phase 1 data informed the questionnaire used for the Phase 2 survey. In Phase 2, data were collected using a postal survey of 651 PHPs. Finally, Phase 3 consisted of additional interviews conducted with 24 more non-adopters of the standards. The quantitative data collected in Phase 2 indicated how widely the themes elaborated in the in-depth interviews were spread across different groups of responders. The Phase 3 qualitative data provided deeper understanding of non-adopters’ perspectives. Focusing on experiences and attitudes of non-adopters, these data provided an opportunity to go back to results of previous phases of data collection and challenge earlier findings.

An explanatory case study [32] was developed for each of the three cases (private pharmacies, private dental practices and private specialist practices). These were then combined to draw a single set of conclusions regarding the situation in the RS and that could be applicable to other health systems. This multiple case study design [32] was appropriate due to the nature of the research question. Each of three cases was first analyzed separately. They were then compared in order to identify the differences observed among the PHPs’ adoption rates of the certification standards. Combining multiple individual cases to identify convergent evidence results in a holistic understanding of common facts and conclusions.

### Qualitative data collection

**Fig. 3:**
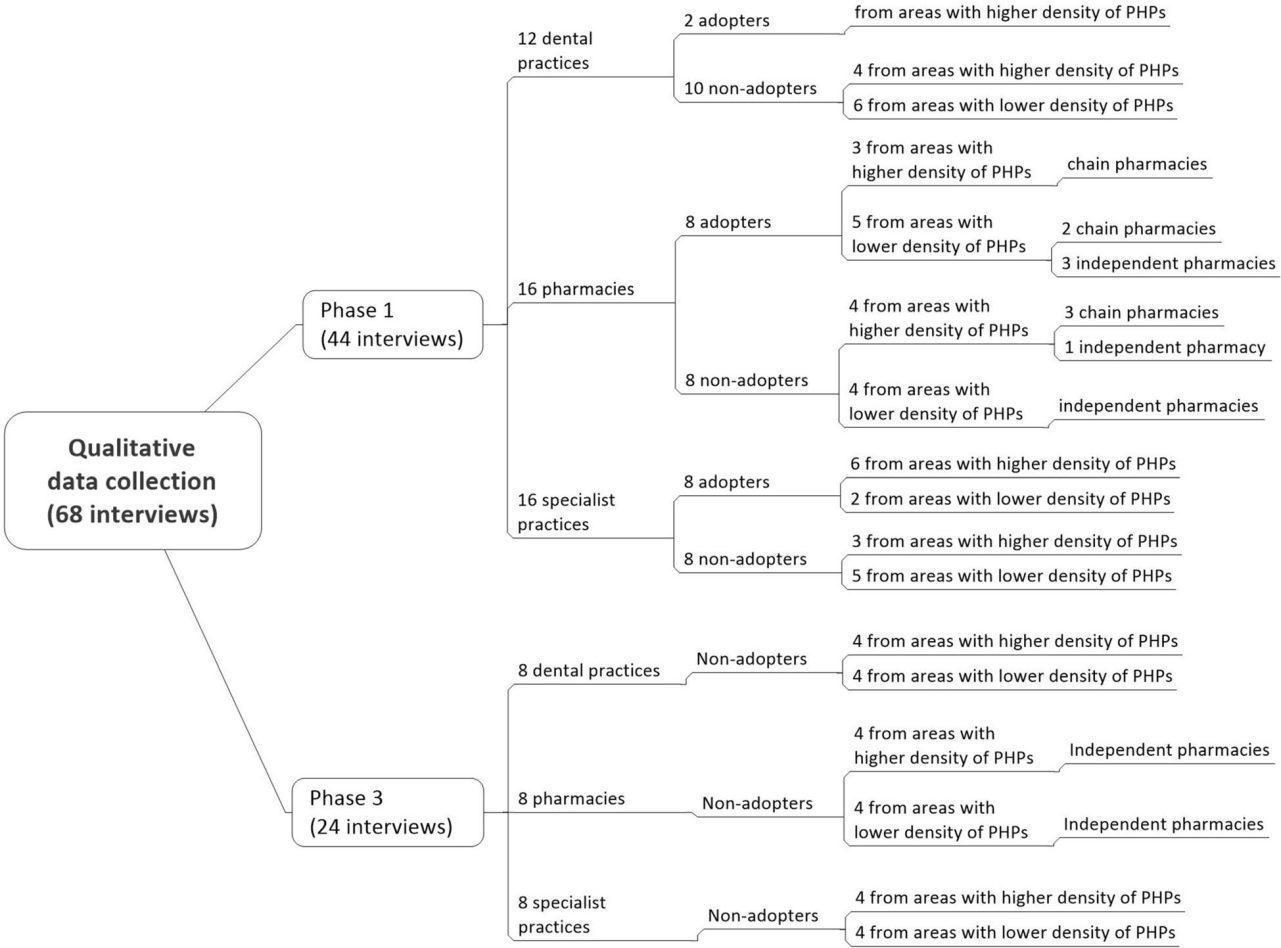
Structure of the samples for qualitative data collection

Interview guides and informed consent forms were developed specifically for the study. The interview guides for adopters of standards (see Additional file 1) and for non-adopters of standards (see Additional file 2) were used for all three types of the PHPs in Phases 1 and 3 of data collection. These interview guides were structured around the eight variables of interest from diffusion of innovation theory. In both interview phases, stratified purposeful sampling for selection of interviewees was applied at four levels: type of provider, status of innovation adoption, density of PHPs in the area and ownership of the pharmacy (Fig. 3). Publicly available registries were used to identify providers fulfilling the inclusion criteria [23, 33]. As data saturation frequently occurs within 12 interviews [34], we estimated that in Phase 1, a sample of 12–16 informants of each type of PHP would be sufficient to reach saturation. We interviewed an equal number of adopters and non-adopters among pharmacies and specialist practices; this proved impossible with the dental practices due to the small number of certified practices. The sample successfully captured major variations among the pharmacies, dental practices and specialist practices (the first stratification level), necessary for confirmation or rejection of the research hypotheses. The additional sampling strata primarily served to increase variation within the sample; some of the variations emerged later in the analysis.

The non-adopter group interviewed in Phase 3 was expected to be more homogenous than the sample of PHPs interviewed during Phase 1. This homogeneity allowed us to reach the data saturation point in Phase 3 more rapidly than in Phase 1. We sampled 8 informants per type of PHP for the interviews in Phase 3. PHPs interviewed in Phase 1 were excluded from participation in Phase 3.

Providers were initially contacted by telephone to confirm their willingness to participate in an interview. Those who agreed were sent follow-up information by post, including more details on the research and a reminder of the time and location of the interview. Interviewees confirmed their voluntary participation in the research by signing informed consent forms prior to the start of the interviews. When an interviewee’s agreement was provided, the interview was recorded with a digital recorder. The recordings were transcribed by the team into Microsoft Word documents. In cases when an interviewee did not agree to be recorded, detailed notes were taken.

The semi-structured interviews were conducted in the Serbian language at the providers’ premises. The member of the research team employed in the ASKVA (SS) did not participate in data collection. All interviewees were asked the questions directly from the interview guide and in the same sequence, but the interviewers also probed inductively to get further information. The sample sizes of 44 interviews in Phase 1 and 24 interviews in Phase 3 proved sufficient to reach the saturation point for all three types of PHPs.

### Quantitative data collection

The quantitative data collection questionnaire specifically developed for Phase 2 of this study covered the selected eight variables from the diffusion of innovation theory (Table 3) [29]. Given the scarcity of publications on this type of diffusion research (Table 2), we were unable to identify a previously validated questionnaire for use in the survey. In the development of the survey questions, however, we drew on examples of validated questionnaires from other studies [35–37].

We took two additional steps to ensure the face validity, readability, consistency and relevance of the questions asked by the survey. The questionnaire was iteratively reviewed by members of the research team and six external experts (two representatives of local PHPs, two representative policy makers and two quality experts), in order to improve formatting, word choice and grammar. The questionnaire was further refined through pilot testing with 22 PHPs, whose responses were used to test the internal reliability of the questionnaire and feasibility of survey administration. In its final form (Additional file 3), the questionnaire consisted of 49 closed-ended questions in the Serbian language. The questionnaire form included no individual identifiers, but the first page of the questionnaire asked respondents to provide basic professional data (e.g. type of provider, number of employees, and certification status). The questions began with the more demanding (Likert scale items), followed by easier to respond to questions with yes/no answers. The questionnaire was created to be a self-administered, paper-and-pen-based tool. Identical questionnaires were used for surveying all three types of PHPs.

The population for the study consisted of pharmacies, specialist practices and dental practices registered in the RS before May 1, 2016 [33]. We used census sampling, targeting all registered pharmacies, specialist practices and dental practices. Pilot respondents were excluded from the sample. Both adopters and non-adopters of the standards were included in the survey. Anonymity of participants was further ensured by the provision of identical sealable pre-addressed return envelopes that were provided together with the questionnaire.

### Data analysis

Thematic analysis of the qualitative data collected in interviews involved mapping the primary data to the eight variables from the diffusion of innovation theory (Table 3). Interview transcripts in Serbian were read through and an initial code list was developed. The codes were repeatedly reviewed and clarified within the research team, until agreement on their consistent application was reached. Coding of the interview data was done independently by two members of the research team (JN and SS) for all interviews. The codebook was continuously updated while the Phase 1 interview data were coded. The same codebook was then used for coding the data from Phase 3 interviews. For every third interview, coding discrepancies were discussed and resolved by the coders. Interview transcripts were uploaded in NVivo 10 software package to facilitate data coding and retrieval. Inter-coder agreement was assessed by calculating Kappa scores for double-coded transcripts [38–40] by running a “Coding Comparison” query. The overall Kappa scores indicated a very good level of inter-coder agreement (Kappa = 0.80 in Phase 1, Kappa = 0.86 in Phase 3) for all nodes. The interview data were first analyzed at the individual provider type level (within-case analysis) before proceeding with cross-case comparisons. Verbatim interview quotes were made anonymous by assigning codes that consisted of specification of provider type (DP = dental practice, PH = Pharmacy or SP = specialist practice), interview number, standards adoption status (C = certified or NC = non-certified) and, for the pharmacies, organizational status (CH = chain of pharmacies or IND = independent pharmacy). The interview quotes for this publication were translated into English by the authors.

Each postal questionnaire was numbered before its data were entered into a Microsoft Excel form. A data entry operator used a list of pre-defined numerical codes. A random check of 10 % of the data entered was performed to verify accuracy. Data were than imported to SPSS 16 for quantitative analysis. This began with identifying the number of members of the sample who did return the survey. Descriptive statistical analysis was provided for all variables, including frequencies, means, standard deviations and ranges of scores. Principal Component Analysis was conducted on the 40-item scale to reveal the factorial structure of the scale of perception of the standards’ properties [41]. The scale initially consisted of eight subscales corresponding to the selected variables from the diffusion of innovation theory (see Additional file 3). The oblimin rotation method was used in the final selection of factors. For the scale and subscales, we computed Cronbach’s coefficient alpha, the most commonly-used type of internal consistency reliability measure. The Chi-square test was used to determine significant differences in the responses of different types of PHPs. *P*-values < 0.05 were considered statistically significant. The one-way ANOVA was used to examine differences in perception of standards’ properties among different types of PHPs and among adopters and non-adopters of the certification standards [41]. The factor analysis is not presented in detail for reasons of brevity, as it confirmed results of the descriptive statistical analyses.

The research team enhanced its overall objectivity in data analysis by including a member (BN) from another country. Neither quantitative nor qualitative data were prioritized in the analysis. Using the same eight variables from the diffusion of innovation theory for both the interviews and the questionnaire enabled comparison, triangulation and integration of results from all three phases of data collection. Triangulation and integration was accomplished through a narrative, weaving approach [42], writing qualitative and quantitative findings together on element-by-element basis. There was a noteworthy level of coherence between the qualitative and quantitative findings and across the three phases of data collection. No findings appeared to contradict or conflict. Throughout the analysis, no individual piece of evidence was considered to be more important than others.

## Results

In Phase 1 and Phase 3, 68 representatives (owners or managers) of PHPs were interviewed, representing 20 dental practices, 24 pharmacies and 24 specialist practices. Of these, all the pharmacies, 45.8% of the specialist practices and none of the dental practices had contracts with the Health Insurance Fund of the RS (RS HIF). About two-thirds (67.6%) of interviewees agreed for their interviews to be digitally recorded.

For the postal survey in Phase 2, 651 private providers were contacted and 223 responded, a response rate of 34.4%. The response rate was highest among specialist practices (53.0%) and lowest among pharmacies (27.1%). One quarter of the respondents (25.1%) had already adopted the standards and certification was ongoing for another 28.7% of respondents, while 46.2% of respondents were non-adopters (Table 4). The lack of dental practice adopters among the respondents was in concordance with the low number of certified dental practices in the RS health system. All pharmacies and 34.1% of the specialist practices had contracts with the RS HIF.

**Table 4:** Characteristics of respondents to the survey

Respondents	Pharmacies	Specialist practices	Dental practices	Total
	*n* (%)	*n* (%)	*n* (%)	*n* (%)
Adopters	44 (42.7)	12 (27.9)	0 (0.0)	56 (25.1)
Certification is ongoing	31 (30.1)	16 (37.2)	17 (22.1)	64 (28.7)
Non-adopters	28 (27.2)	15 (34.9)	60 (77.9)	103 (46.2)
Total	103 (100.0)	43 (100.0)	77 (100.0)	223 (100.0)

The final scale, on perception of the standards’ properties, revealed by the Principal Component Analysis, consisted of six subscales. Some of the initial components did not form separate factors (certification standards characteristics, interpersonal communication channels and persuasion), while unfavorable attitudes towards certification standards formed a distinct component from the favorable ones. Overall, the six-factor model explained 63.5% of variance in the set of 40 items, as shown in Table 5. The contribution of the first factor (relative advantages of standards; perceived benefits) was 35.0%, the second factor (influence of chambers) contributed 8.5%, the third (influence of professional associations) contributed 6.8%, the fourth (perceived disadvantages of standards) contributed 5.9%, the fifth (observability) contributed 3.7% and the sixth factor (availability of information on certification) contributed 3.6%.

**Table 5:** Results of one-way analysis of variance, by provider types

Subscales	Type of private health care provider	n	Mean	Standard deviation	F (p)
Relative advantages (perceived benefits)	Pharmacies	101	24.59	7.82	35.906 (.000)
	Specialist practices	43	20.88	8.96	
	Dental practices	74	14.32	7.44	
	Total	218	20.38	9.12	
Collective innovation-decision (influence of chambers)	Pharmacies	98	17.67	5.52	9.533 (.000)
	Specialist practices	40	13.77	6.52	
	Dental practices	70	14.49	5.57	
	Total	208	15.85	5.97	
Collective innovation-decision (influence of professional associations)	Pharmacies	99	18.47	5.18	22.972(.000)
	Specialist practices	40	14.72	7.28	
	Dental practices	72	12.78	4.89	
	Total	211	15.82	6.10	
Relative advantages (perceived disadvantages)	Pharmacies	102	19.93	4.50	5.646 (.004)
	Specialist practices	43	22.19	3.57	
	Dental practices	76	21.63	4.34	
	Total	221	20.95	4.37	
Observability	Pharmacies	99	12.60	4.44	9.339 (.000)
	Specialist practices	38	10.42	4.55	
	Dental practices	69	9.74	4.29	
	Total	206	11.24	4.58	
Innovation-decision process: knowledge (availability of information on certification)	Pharmacies	102	16.17	3.62	31.068 (.000)
	Specialist practices	42	13.90	4.75	
	Dental practices	71	11.30	4.05	
	Total	215	14.12	4.53	

The factors influencing the adoption process are described using the eight variables from the diffusion of innovation theory as the framework for grouping the results.

### Perceived attributes of the standards: Relative advantages

Differences in perceptions of the benefits of the standards were mostly noted among those PHPs who adopted the standards (Table 6). Many adopters from the pharmacies (particularly those from pharmacy chains) and some interviewees from certified specialist practices felt that the introduction of standards was beneficial to their organizations, as it facilitated management and increased professional confidence and safety. For example, one respondent stated:

*In all our pharmacies, the work is performed according to the same procedures, which makes it easier to control the work and employee’s performance.* PH11/C/CH.

**Table 6:** Synthesis of interviews and survey findings on providers’ perceptions of safety and quality standards’ attributes

Type of private health care provider	Adoption of standards	Relative advantages		Observability
		Perceived benefits	Perceived disadvantages	
Pharmacies	Adopters	Significant: ↑management ↑ professional confidence ↑ safety	Insignificant	Observable effects: Inspectors RS HIF Patients
	Non-adopters	Insignificant	Significant: ↑ expenses ↑ administrative burden ↑ loss of time	No observable effects
Specialist practices	Adopters	Uncertain significance: ↑ infection control ↑ safety	Significant: ↑ expenses ↑ working hours	Few observable effects: Owner of practice Staff
	Non-adopters	Insignificant	Significant: ↑ expenses ↑ administrative burden ↑ loss of time	No observable effects
Dental practices	Adopters	Insignificant	Insignificant	No observable effects
	Non-adopters	Insignificant	Significant: ↑ expenses ↑ administrative burden ↑ loss of time ↑ disruption of service provision	No observable effects

Note: ↑ indicates improvement (perceived benefits) or increase (perceived disadvantages)

Interview and survey findings alike confirmed that dentists did not perceive substantial advantages in adoption of the standards. The majority of survey respondents from dental practices (68.9%) did not perceive that the introduction of standards could facilitate management of their practices. This same attitude was strongly or partially expressed by only 28.1% of respondents from specialist practices and 25.0% of respondents from pharmacies (Chi square = 52.5, *p* = 0.000).

Regardless of the type of provider, the non-adopters interviewed strongly emphasized disadvantages of the introduction of standards such as associated expenses, increased administrative burden and disruption of service provision. A synthesis of findings on the perceived disadvantages, based on triangulation of qualitative and quantitative data and differentiated between provider types, is presented in Table 6. All types of providers questioned the suitability of a single set of standards for different organizational structures and sizes. With the exception of chain pharmacies, PHPs stated they would not participate in certification without regulatory enforcement.

Some interviewed adopters from specialist practices stressed disadvantages of the standards. They believed that it took long to PHPs to introduce the standards, that it consumed too much professional time and that the standards’ requirements were overly general and not sufficiently adapted to the different medical specialties’ services. A few interviewees from both dental and specialist practices questioned why certification was not applied in the same way across private and public health care providers. The latter group was seen as being protected against negative financial consequences (including payment of fines) of not complying with the standards. One interviewee stated:

*Public health care institutions are protected and they behave as they like. Many do not fulfil the certification conditions, and yet they have completed certification. Certification and its conditionality ought to be the same for all.* SP12/C.

Disadvantages of the standards were highlighted in survey responses from all three types of the PHPs. The opinion that there were too many requirements in the standards was strongly or partly expressed by 81.7% of respondents. Further, 79.3% of respondents believed that standards had to be better tailored to the type and size of provider, although they agreed that the standards as such could be implemented (77.2%). One interviewee also expressed a concern that the local standards were, when compared to internationally recognized standards, insufficient:

*I saw no benefit….I will go back to the Joint Commission International’s standard, which is really expensive, but enables me to bring foreign patients here….What has this certification similar to offer? Nothing.* SP20/NC.

Differences in respondents’ perception of the complexity of the standards’ requirements were evident. Just 36.1% of respondents from dental practices considered the requirements of standards to be clearly defined, while this opinion was strongly or partly expressed by 74.0% respondents from pharmacies and 75.7% from specialist practices. Compatibility of standards with existing values, past experiences and needs was questioned by some, including this non-adopter from a dental practice:

*They [standards] are too broad and too demanding to be dealt with in 10–15 days. Even one month is not sufficient time. That’s the major problem. Standards should be simplified….With less demanding standards, certification might have been finished so far.* DP19/NC.

Non-adopters from dental practices also saw the standards as imposed on, instead of negotiated with, the profession. Some felt that the standards were too stringent to the current level of health system development:

*We are not a developed European Union country….It is not the right moment for such initiative.* DP3/NC.

### Perceived attributes of the standards: Observability

Regardless of the type of provider, study participants perceived that results of the standards adoption were not made sufficiently visible to, for example, patients, inspectors, the RS HIF, and the line Ministry. Less than a quarter (22.9%) of survey respondents agreed to some degree that patients might observe differences in functioning between PHPs that introduced the standards and those that had not. Only one-third of respondents strongly or partly perceived that the adopters of standards were recognized by the public as examples of good practice (33.7%); similar proportions believed that the line ministry (33.8%) and inspection teams (39.0%) positively valued such providers.

Some differences in perception of observability were noted among those PHPs who had adopted the standards (Table 6). Adopters from pharmacies reported that the adoption of standards was observable by inspectors, RS HIF, and patients, while adopters from specialist practices reported only observability within the practices. A majority of dentists did not consider observability to be an attribute of the certification standards. Adopters confirmed in interviews that observability of compliance with the standards was missing:

*People don’t know that we have been certified; they don’t even have information that somebody was certified, because it is a closed circle between us and those who have certified us….Let’s go with certification to media, let’s raise it to a level that an ordinary patient is familiar with certification and that he can say ‘From now on, I will choose a certified provider.’* DP12/C.

### Communication channels: Mass media

The PHPs’ main sources of information on the standards were peers, the ASKVA, professional associations and health chambers. Table 7 presents a synthesis of interview and survey findings on influential sources of information, differentiating between provider types and based on triangulation of qualitative and quantitative data. The ASKVA provided information through its web site and official communications with PHPs. The majority of interviewees from the certified pharmacies reported that they actively searched for information and mostly got sufficient and correct information about the standards from sources they considered trustworthy, namely the ASKVA and the Pharmaceutical Society of the RS. One interviewee reported:

*We did not hesitate to ask ASKVA about anything that was not clear regarding standards and their application, so this was really an active involvement of me and other pharmacists, according to our responsibilities.* PH15/C/CH.

**Table 7:** Synthesis of interviews and survey findings on influential sources of information and frequent communication channels

Type of private health care provider	Adoption of standards	Sources of information (ranked by influence)					Communication channels (ranked by frequency of use)				
		ASKVA	Peers	Professional association	Public Health Institute	Chamber	Internet	Professional meetings	Training events	Interpersonal communication	Official correspondence
Pharmacies	Adopters	***		**			✦✦✦	✦✦	✦		
	Non-adopters	**	***	*			✦✦✦	✦✦	✦		
Specialist practices	Adopters	***	**		*		✦✦		✦✦✦	✦	
	Non-adopters	**	***	*				✦✦		✦✦✦	✦
Dental practices	Adopters	***					✦✦✦		✦✦	✦	
	Non-adopters	*	**			***		✦✦		✦✦✦	✦

Ranking of information sources: *** the most influential source; ** source with secondly ranked influence; * source with thirdly ranked influence

Frequency of communication channel use: ✦✦✦ the most frequently used; ✦✦ frequently used; ✦less frequently used

Television, radio, press, professional magazines, the Official Gazette of the RS, and the line ministry were not identified (in either interviews or in the survey) as important information sources for any type of the PHPs.

### Communication channels: Interpersonal channels

Direct contact with peers was commonly used by all interviewees, regardless of provider type or adoption status, to obtain information on the standards, as described by one interviewee:

*I asked for advice from colleagues who had already been certified...Then I spoke with the colleagues who were in the certification process at the same time or had started a bit earlier. We were constantly in touch.* SP8/C.

Interpersonal communication was used more by non-adopters, often serving as their sole channel of communication. Some level of competition, vanity or jealousy was occasionally present when peers were providing information on standards. A non-adopter from a dental practice reflected on this:

*We don’t ask for information from others; we all think we know everything…Does a dentist, who has the right information, want to share it with peers?* DP4/NC.

A significant majority of survey respondents (83.6%) reported turning to peers to obtain information about the certification standards; this included both direct interpersonal communication and interactions at professional meetings. A majority of respondents (85.2%) asked for peer advice in relation to the standards, while 47.4% of the respondents stated that the opinions and actions of their peers influenced their decision on whether to adopt the standards. Just over half of pharmacies (52.6%) and specialist practices (51.4%) used contacts with adopters to obtain information; among dental practices this dropped to 27.4% (Chi square = 10.5, *p* = 0.005). Information obtained from the Public Health Institute of the RS through training events and direct support in complying with the requirements of the standards was important to more specialist practices (48.6% of respondents) and dental practices (43.9%), compared with pharmacies (14.1%).

### Innovation decision process: Knowledge

The majority of interviewees from the pharmacies demonstrated substantial knowledge about the certification standards for pharmacies, as well as quality and safety improvement processes. However, non-certified interviewees from specialist practices and dental practices demonstrated a significant level of misinformation about the standards requirements, certification-related costs, the external assessment process, the purpose of certification, the ASKVA ownership and deadlines for standards adoption. A majority of them had not read the certification standards. The levels of misinformation and lack of knowledge on standards were most evident among dental practices. A non-certified dentist described his understanding of what it would entail:

*As soon as you enter the practice in the morning, you need to fill in ten pages of documentation.* DP18/NC.

Some dentists suggested that misinformation on certification standards was, to a certain extent, deliberately spread by illegally practicing dentists, who falsely presented the issues that were, in reality, not obstacles to the certification of dental practices:

*There was a revolt on certain issues, such as disposal of the medical waste, infection control, filling out the questionnaire and patients consent…These were presented as the problems by some of us who hide as they do not have basic documents, do not register employees, have no fire extinguishers, have no work permits…They do not wish to fulfill these conditions. I think this is a false presentation of the certification problem.* DP11/NC.

### Innovation decision process: Persuasion

Regardless of the type of provider, the non-adopters who were interviewed expressed unfavorable attitudes towards the standards. Many non-adopters believed that certification would not lead to improvements in safety and quality of health care. Only certified pharmacies expressed favorable attitudes towards the standards.

Half of survey respondents (49.6%) anticipated that the most important gain from complying with the standards would be advantages in contracting with the RS HIF. Pharmacies expected more gains than other two types of PHPs. The different expectations of different provider types were statistically significant when considering advantages in contracting with the RS HIF (Chi square = 33.9, *p* = 0.000), as well as when considering gains related to patient satisfaction, possibility of gaining additional patients, gains in professional status and gains related to staff satisfaction. The anticipated advantages of contracting with the RS HIF were often not achieved even after introduction of the certification standards; only 23.5% of respondents to the survey reported gains. Interviewees who adopted the standards did, however, mention some other gains. For example:

*We are not only monitoring the quality of the hygiene in the pharmacy now, we are monitoring customer satisfaction.* PH11/C/CH.

**Fig. 4:**
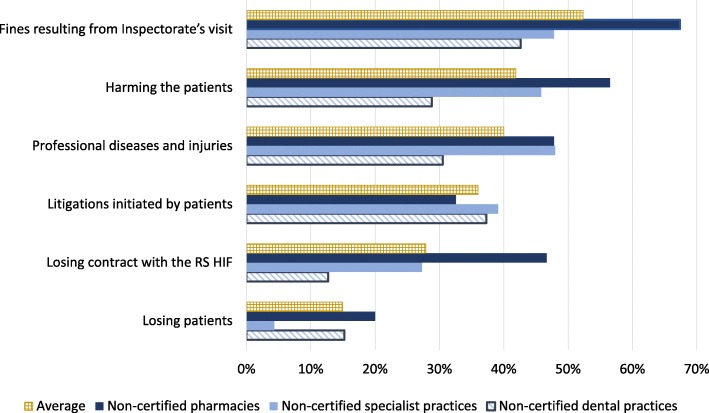
Non-adopters’ perceptions of risks related to delaying introduction of safety and quality standards. Legend: Subset of data collected through the survey

Non-adopters among all three types of PHPs perceived two important risks that could be mitigated by the certification process (Fig. 4): the risk of paying fines in response to an inspector’s visits (52.3%), and risk of harming patients (41.9%). Survey findings also suggested that the non-adopters among pharmacies were significantly more inclined than other non-adopters to perceive that certification can mitigate the risks of fines and of losing a contract with the RS HIF (Chi square = 14.2, *p* = 0.001). None of the PHPs types perceived that risk of losing patients could be significantly mitigated by adoption of the standards given the limited visibility of the certification status to the patients (Table 6). There were no statistically significant differences among the different types of PHPs with regards to their perceptions of the potential of the certification process to mitigate risk of litigation nor of professional diseases and injuries.

### Innovation decision process: Decision

The certification-related costs and the lack of adequate information on the certification process were the main factors for many non-adopters among pharmacies delaying the introduction of the standards. Most were aware that they would have to introduce standards eventually, and some stated they were just waiting for the ASKVA to schedule the external assessment. During Phase 1, the majority of pharmacy adopters confirmed the main motivators for adopting the standards: knowledge about benefits stemming from the standards, legal reasons arising from mandatory certification, the risk of losing contracts with the RS HIF, and the risk of losing patients. One interviewee stated:

*When we applied for the Health Insurance Fund contract…(I think that it was a contract for 2013/2014), among other documents they asked for confirmation that we have applied for certification. Considering all the risks, the contract with the Fund was dominant.* PH14/C/IND.

Two principal motivations for delaying certification were reported by non-adopters from specialist practices: the high price of certification and the lack of perceived professional benefits related to standards introduction. The legal obligation, on the other hand, was the main motivator for adopters among specialist practices, although the risk of losing contract with the RS HIF was also occasionally mentioned.

Among the dental practices, the main motivators mentioned by interviewees were legal reasons, personal reasons and professional status. The main motivation for non-adoption of the standards by dental practices, on the other hand, was a perception that certification would have significant negative consequences (such as a reduced number of patients and lower income) and that the practice would become less competitive in the market. In the words of an interviewee from a non-certified dental practice:

*Would this later dictate prices of our services?...If money is required for the certification process, I have to take it from the patients. Do they have more money? We need to adapt to living standards in our country… Should I have a certified practice and sit alone [without patients] in an empty practice?* DP16/NC.

### Social system: Collective innovation-decision

**Fig. 5:**
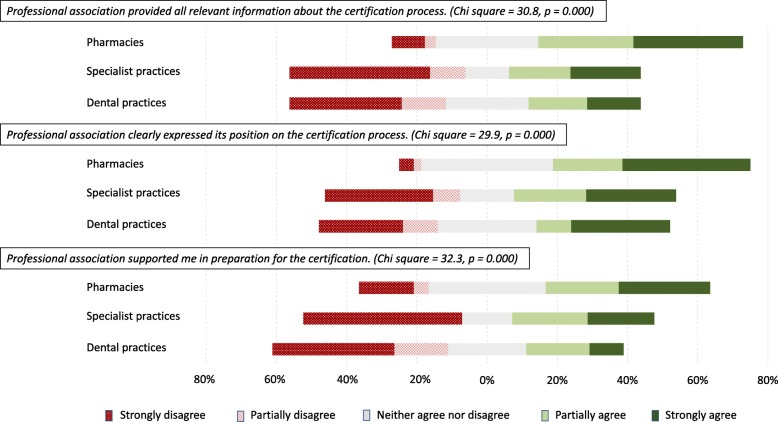
Perception of professional associations’ influence on adoption of the standards. Legend: Subset of data collected through the survey

The study’s survey findings suggested that the pharmacists’ professional association was more influential in the innovation-decision process than the professional associations for doctors and dentists. The Pharmaceutical Society of the RS provided more information and support to its members; it also publicly expressed its position on the standards more clearly than other professional associations (Fig. 5). Interviewees also emphasized that the Society had a positive attitude towards the certification standards. It both offered support to its members in fulfilling their legal obligation and supported the development of operating procedures required by the standards, sharing templates with its members. One pharmacy representative stated:

*The Society was of great assistance to me; I have got all procedures from them. If I was not a member, and if I did not have access, I do not know how this could be done. Those who are members of the Society have 50% of work done for certification. Whatever is not clear, I go to them and get assistance again.* PH5/NC/CH.

In contrast, none of the professional associations of dentists mentioned by the interviewees was interested in certification, nor did they make public statements on the certification process. Among the interviewees from the specialist practices, about half stated that they were unaware of the official position of the Association of Private Medical Doctors of the RS on the standards. This was further explained by a non-adopter from a specialist practice:

*The opinions and attitudes in the Association of Private Medical Doctors are very much different; they are only presented in informal conversations. Officially, certification is supported; unofficially they say that for the time being there is no need to go into the certification process and that we should wait.* SP23/NC.

The vast majority of respondents to the survey from dental practices (92.8%) confirmed using their chamber as a source of information on certification standards. Only 39.4% of pharmacies and 21.6% of specialist practices reported similar use of their respective chambers (Chi square = 65.5, *p* = 0.000). Correspondingly, the majority of the interviewees from specialist practices and pharmacies were not aware of the official position of their chambers toward introduction of the standards and reported that the chambers had not influenced their choices.

For non-adopters among the dentists, the Chamber of Dentists of the RS was the most influential source of information about the standards (Table 7). The Chamber, although perceived in the beginning of the process as a platform for organizing active resistance against the introduction of standards, did not come up with an official position on certification. Lack of clarity on the Chamber’s position was evident throughout the interviews. Some interviewees perceived that the Chamber of Dentists of the RS supported certification, while the majority of non-adopters regarded the Chamber to be officially against introduction of standards from the beginning until the time of interviewing. One interviewee commented:

*We had two meetings on certification within the Chamber and did not agree to it. The President of the Chamber requested for the certification to be postponed and abolished… Nothing could be changed. There was no response from the Ministry.* DP19/NC.

**Fig. 6:**
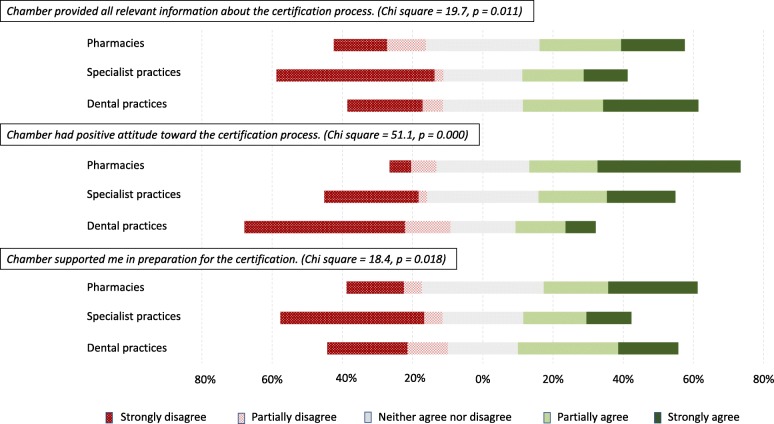
Perception of health chambers’ influence on adoption of the standards. Legend: Subset of data collected through the survey

This lack of clarity was confirmed by the survey. The majority of dental practice respondents reported that the Chamber of Dentists of the RS had a negative attitude toward the certification process (Fig. 6). The support of the relevant chamber for certification, including information provision, was less available to specialist practices than it was to pharmacies and dental practices.

## Discussion

In the RS, pharmacies adopted quality and safety improvement standards at the fastest rate; specialist practices have been slower, and the slowest rate of all was among dental practices. This study sought to understand why safety and quality standards for certification were adopted more rapidly by some PHPs than by others. To our knowledge, this is the first study to examine differences in adoption of mandated health care quality improvement initiative in various parts of a health system.

The findings of the study indicate that the rates of adoption varied among different types of PHPs due to: different levels of availability of information on the standards and implementation process among the different groups of providers, influencing the knowledge stage of the innovation-decision process; different levels of concern about possible negative financial consequences, influencing the persuasion stage of the process; and, influencing the decision stage, different levels of support from professional associations support for introduction of the standards and differences in perception of health chambers’ attitudes toward the standards.

The diffusion process is founded on an exchange of information through which a new idea is communicated to others [29]. In this example from the RS, the availability of appropriate information influenced the adoption of the safety and quality standards by PHPs. In all three cases, the ASKVA was reported to have been relatively passive in the provision of formal information on standards to the PHPs. A systematic and organized information campaign to provide correct and timely information to ASKVA’s potential clients was missing. A common finding across all three cases was that the diffusion of information on standards among the PHPs had been generally unplanned, informal, decentralized and mediated by peers. Another common finding in the three cases was that interpersonal influences through informal social networks (which are identified in literature as dominant mechanisms for diffusion of innovation in service organizations [30]) were the principal mechanism for spreading information about the standards. Interpersonal communication networks with close peers were particularly important for conveying evaluative information about the innovation that reduced uncertainty about expected consequences [29].

Communication patterns among the PHPs confirmed earlier findings from another study [43] that different categories of health care professionals were likely to communicate informally with members of the same professional group, while their connections with other professions were sporadic and fragmented. PHPs tended to informally communicate within narrow disciplinary communities of practice. Such boundaries between informal networks of different professions slowed the spread of information on quality and safety standards among three types of providers [44]. Non-adopters were more likely to seek out collegial support to confirm their attitudes and to justify a decision to postpone the adoption of standards. Negative influences were often informally conveyed by non-adopters to other PHPs in the same social sub-system [45]. The highest level of misinformation was found among dental practices, contributing to their lower rate of adoption of the standards.

Undesirable outcomes, such as possible negative financial consequences related to fines or loss of contracts with the RS HIF, could be avoided by adopting the standards. All pharmacies and a majority of specialist practices had contracts with the RS HIF. The RS HIF does not purchase services from private dental practices. Even though the RS HIF did not require certification as one of the contracting criteria in the 2014–2016 period, pharmacy owners reported feeling at risk of losing their contracts with the RS HIF. This risk was less important to the specialist practices and was not considered significant among the dental practices. Other studies have shown that health care providers will not work to meet safety standards if they do not face substantial threats to their business – as when a purchaser shifts funding away – as a result [46]. Across all three cases, the study found non-certified PHPs were aware of the existence of risks related to non-adoption of the standards. However, they were willing to face them by delaying participation in the certification program. Negative results, such as a loss of contract with the RS HIF, might or might not occur. Under these uncertain circumstances, PHPs’ motivation to adopt the standards was not strong. The risk of fines imposed by inspectors was the only factor that PHPs reported was sufficiently severe to influence their decision on standards adoption. Perceptions of these risks were important in developing PHPs’ attitude towards the innovation, but also in influencing their compliance with the standards.

Rogers [29] posits that the greater the perceived relative advantage of an innovation, the more rapid its rate of adoption; similarly, the easier it is for individuals to see results of an innovation, the more likely they are to adopt it. Regardless of provider type, non-adopters did not perceive advantages to compliance with the standards. Social and professional prestige were also not crucial influences in adoption of the standards in any of the three cases. This is consistent with another previously noted observation: that relative advantages of preventive innovations can be perceived as delayed rewards [29]. All types of PHPs reported relative disadvantages related to adoption of the standards, a finding that was consistent with observations from other countries [15]. All types of PHPs also perceived the standards as complex and incompatible with the needs of providers and the context. This perception was important, serving as an argument that mobilized discussions among peers against support for the certification program. The lack of visibility of certification status seemed to discourage discussion about the standards within peer clusters, contributing to slow diffusion of the innovation [29].

By mandating the adoption of standards, a system can exert pressure on an individual to acknowledge the relative advantages of an innovation, particularly in the case of preventive innovations [29]. When innovations in health care are externally mandated, however, the decision to adopt originates externally from the organization responsible for implementation of the innovation [24]. If meaningful enforcement mechanisms are lacking in the health system, compliance with the legal obligation effectively becomes voluntary [47].

In countries such as Bosnia and Herzegovina (and many other countries in transition) formal legal systems may be weak and laws may exist on paper but go unenforced [48]. Gaps between what is written in the regulation and what happens in practice are evident in the case of mandated safety and quality standards in the RS health care system. Traditional methods of implementing regulations, such as administrative searches, inspection and licensing [26] were available to health authorities in the RS. However, as a significant share of public health care providers had not yet adopted the safety and quality standards, the authorities had not imposed sanctions on non-adopter PHPs. Imposing fines, though known to influence relative advantages and affect innovation adoption rates [29], was not fully utilized. Effectively, the responsibility for deciding to adopt the standards was left to the individual provider. What was originally considered an authority innovation-decision thus evolved instead into a contingent innovation-decision involving two or three sequential decisions. The first decision was an authority innovation-decision made at the RS health system level by policy makers with power and technical expertise. The second tandem decision was made by the governing bodies in health chambers and PHPs professional associations; this represented a collective innovation-decision. Because sanctions were not being imposed for nonadherence, the final tandem decision was made by each individual provider independently of the decisions of other PHPs; this represents an optional innovation-decision.

The goals of the various professional associations of providers in the RS differ according to the needs of their members. Some of their interests might include strengthening the position of the organization or gaining additional membership. The more an association perceived that certification according to the standards aligns with its interests, the stronger that association’s support for the introduction. As the Pharmaceutical Society of the RS supported the certification process, compatibility undoubtedly existed between the process and organizational goals of the Society. As a result, the Society became a champion of the process, providing private pharmacies with continuous access to credible information about the standards. The Society also organized training for its members, which helped to alleviate pharmacy representatives’ concerns during the innovation decision process and assisted in complying with requirements of the standards [30]. Social interactions among pharmacists at professional meetings and training events were reported by respondents to have been important for creating early awareness about the innovation [43]. Pharmacy owners demonstrated more awareness of the purpose of the standards than the other two types of PHPs; they actively searched for information and benefited from efforts led by the professional association of pharmacists.

The medical profession faces difficulty when trying to harmonize numerous specializations’ interests into a unified position of a professional association or chamber; as a result, their positions on the innovation of quality and safety standards were far more varied than the pharmacists’. Unlike the two other chambers, a majority of members of the Chamber of Medical Doctors of the RS are in fact employees of the public health care facilities. They showed little interest in improving quality of health care services among PHPs. Among the dentists, the Chamber of Dentists of the RS emerged as the sole collective actor in the profession’s debate on the standards, as the dentists’ professional associations are relatively weak. The Chamber questioned the need for mandatory introduction of the standards, considering them to be more an inspection, rather than a quality improvement, process [49]. The Chamber expressed clear disagreement with the innovation by requesting the line ministry to make compliance with the standards voluntary instead of mandatory. It also questioned the appropriateness of the standards established for dental practices. This not only expressed a difference of opinion on the need for innovative change; it also hindered the introduction of standards and the Chamber lost the opportunity to engage and collaborate with health authorities on strategizing the standards introduction [11].

Increasing the rate of adoption of mandatory quality and safety standards among private health care providers in the RS requires interrelated and interdependent approaches that simultaneously focus on different stages of the innovation-decision process. The mass media have been underused for the knowledge stage. They could have been engaged by health authorities to provide better and ongoing support to the certification process and promote certified public and private health care providers. Publicly promoting early adopters of the standards could make later adopters feel more secure about deciding to enter the certification process. Another strategy to improve knowledge would be encouraging the ASKVA to provide PHPs with more credible and objective information on the standards. This could contribute to reducing the prevalence of misinformation among providers. Professional associations could be encouraged to include health care quality and safety topics in continuing medical education programs; this could raise awareness and increase knowledge on standards, while providing additional opportunities for interpersonal communication among peers who had adopted the standards.

At the persuasion stage, strategies to encourage adoption include setting clear deadlines for compliance by both public and private providers with regulatory requirements and increasing the risk of unwanted events occurring to non-adopters of the standards. Health authorities could better align the certification process with inspection, licensing and other regulatory procedures, contributing to changes in perceptions of the relative advantages of the standards. Penalizing individual non-adopters by inspection would be perceived as an immediate negative incentive; it could be used for public and private health care providers. Another strategy would entail modifying the Health Insurance Fund’s contracting approach to favor providers that have met the standards. This would offer immediate positive monetary incentives for individual adopters of the standards, rather than remaining a delayed reward.

Finally, at the decision stage, social norms and group pressure could be created by (1) continuing to engage with and extend professional associations’ support to the PHPs’ introduction of standards; (2) assuring and demonstrating engagement of health chambers in the certification process; and (3) communicating more with the general public on the purpose and achievements of the certification program. These recommendations could also be relevant to policy makers in other low- and middle-income countries in which the introduction of a compulsory accreditation scheme is being considered.

### Limitations

This study was conducted only with PHPs, although the safety and quality standards are mandatory for both public and private health care providers in the RS. Public health care providers are part of the wider social system in which diffusion of the standards among the PHPs has taken place. Results of the study demonstrated that the behaviors of public providers had effects on the behavior of PHPs. As there were no comparable data from public health care providers, we were not able to compare standards adoption processes between public and private sector providers. Further study is warranted with a broader view that includes public health care providers.

The duration of the study only allowed for the first three stages of the innovation-decision process—knowledge, persuasion and decision—to be examined. Future research should also consider covering the remaining two stages, implementation and confirmation.

The survey questionnaire was developed on the basis of diffusion of innovation theory [29] and it may have failed to elicit influences not congruent with the theory. Also, a relatively limited number of providers responded to the survey. The sample size was sufficient to allow testing of differences between three types of PHPs. Still, given the absence of certified dental practices, and low absolute number of providers in some subtypes, it was not possible to analyze data at subtype level (e.g. dental practices disaggregated by adoption status). More responses, particularly from the adopters among dental practices, could have added important perspectives and balanced the views of this type of PHPs.

More customization of the survey questions to different PHPs might have been possible if it had been administered online. However, given uncertain levels of information technology literacy among PHPs, it was reasonable to administer a postal survey with a limited number of questions, instead of an online survey. The survey results were triangulated with qualitative data from the semi-structured interviews to mitigate these limitations.

The interviewees were purposefully selected to form a representative sample in terms of provider type, standards adoption status and density of the PHPs in the area. However, this sampling strategy could bias the results. To mitigate this risk, we aimed to reach a data saturation point in both Phase 1 and Phase 3 interviews with all three types of the providers. Each of the three interviewers conducted interviews with only one type of PHPs, which allowed each of them to determine the saturation point for the group. The possibility exists that interviewees could have been selective in what they reported, or even that they responded to questions inaccurately, in order to present themselves in the best possible light. Again, the interview results were triangulated with quantitative data to mitigate the social desirability bias.

## Conclusions

This study adds to knowledge on the relevance of the diffusion of innovation theory to quality and safety improvement initiatives in the health sector. The theory proved to be a suitable framework for examining how a decision on adoption of standards-based certification of PHPs was made by various health care providers within the health system of the RS. The results of the study demonstrated that the rate of adoption of standards was higher when: adequate and trustworthy information on the standards was provided in a timely fashion; when non-compliance with regulation was perceived to be associated with significant financial risks; and when authorities engaged with professional associations and health chambers, partnering with them to advocate for adoption of the standards. The experiences from the RS documented in this study illustrate how national health authorities seeking to introduce mandatory standards for private for-profit health care providers can use these interlinked influences to reinforce compliance. Provider adherence with regulation, in turn, will accelerate progress toward achieving universal quality health coverage and increasing equity across the health system.

## Additional files

Additional file 1: Interview guide for the private healthcare providers that have completed certification. Interview guide used for interviews with pharmacies, specialist practices and dental practices that adopted the safety and quality standards. (DOC 56 kb)

Additional file 2: Interview guide for the private healthcare providers that have not completed certification. Interview guide used for interviews with pharmacies, specialist practices and dental practices that adopted the safety and quality standards. (DOC 74 kb)

Additional file 3: Questionnaire for the private healthcare providers. The questionnaire used in self-administered postal survey of pharmacies, specialist practices and dental practices. (DOC 190 kb)

## Abbreviations

ANOVA: Analysis of variance
ASKVA: Agency for Certification, Accreditation and Healthcare Quality Improvement in the Republic of Srpska
C: Certified
CH: Chain of pharmacies
DP: Dental practice
IND: Independent pharmacy
NC: Non-certified
PH: Pharmacy
PHP: Private health care provider
RS HIF: Health Insurance Fund of the Republic of Srpska
RS: The Republic of Srpska
SP: Specialist practice
SPSS: Statistical Package for the Social Sciences
