# Supplementary material for: Introduction of safety and quality standards for private health care providers: a case-study from the Republic of Srpska, Bosnia and Herzegovina
Source: Int J Equity Health. 2018 Oct 5;17:92. doi: 10.1186/s12939-018-0806-0 (PMC6172732; doi:10.1186/s12939-018-0806-0)
Supplement: Supplementary file 1 — Interview guide for the private healthcare providers that have completed certification. Interview guide used for interviews with pharmacies, specialist practices and dental practices that adopted the safety and quality standards. (DOC 56 kb) [file 12939_2018_806_MOESM1_ESM.doc]

### Interview guide for the private healthcare providers that have completed certification

**Introduction, consent and general information**

***Note:***

Start by introducing yourself and the purpose. If this is agreeable to interviewee, proceed to the consent form.

**General information: take notes only, not to be recorded**

1. Type of provider: 1 Pharmacy 2. Dental practice. 3 Specialized doctors practice
2. Location municipality ………………. urban …..rural……
3. No of employees…………
4. Contract with Health Insurance Fund: Yes …No…
5. Status (chain or independent) for pharmacies:………..
6. Years of work experience…. Years in private practice….
7. Position in organization: 1. Owner… 2. Managing director….
8. Membership in professional associations …………..

Request to record interview and make sure the tape recorder is switched on. If not, detailed notes should be taken

**Section 1 Context**

1. What do you think of level of quality and safety of health care services provided in Republic of Srpska?

Probe:

1. How much are you informed about process of certification of health care institutions in Republic of Srpska?
2. What is your opinion – is there a difference between quality of services provided by private and public providers? If so can you explain why?
3. How do you perceive quality of services you provide?

**Section 2: Perceived attributes of innovation**

1. What do you think of quality and safety standards included in certification process?

Probe:

1. Have you or some of your colleagues been involved in development of standards?
2. How relevant are safety and quality standards to the health services provided by your practice/pharmacy?
3. What were the major obstacles in implementation of quality and safety standards in your practice/pharmacy?
4. What benefits of certification have you observed within your pharmacy/practice or within practices/pharmacies of your colleagues, if any?

Probe:

1. Have you observed any drawbacks?

**Section 3: Potential gains vs. fear of consequences**

1. Prior to joining the program, what were your expectations about potential gains in relation with adoption of quality and safety standards?

Probe:

1. Gains in professional status?
2. Gains related to patient’s satisfaction?
3. Gains related to staff satisfaction?
4. Some other gains?
5. Have you considered that there might be some negative consequences if you chose not to adopt the quality and safety standards?

Probe:

1. Higher risk of harming the patients?
2. Risk of losing contract with Health Insurance Fund?
3. Risk of losing the patients?
4. Some other risks?

**Section 4: Communication channels and knowledge about innovation**

1. What type of information about certification was available to you during the process of deciding whether to join the certification program?

Probe:

1. What were the main sources of information (mass media, TV, radio, newspapers, professional magazines, Internet, personal contacts, seminars, professional associations, representatives of health authorities such as Agency staff or health inspectorate)? Were the information appropriate?
2. Which channel of communication you have found to be the most informative?
3. What steps have you taken to find and access relevant information? Please explain.
4. Have you had necessary knowledge and skills to introduce the standards in your practice/pharmacy?

Probe:

1. Have you looked for professional assistance in order to adopt the standards?
2. Have you looked for advices from your peers?

**Section 5: Innovation decision process**

1. What factors influenced you to join the program?

Probe:

1. Could you estimate how long it took you to decide to join the certification program?
2. How much has previous knowledge about certification influenced your decision to join the certification program?
3. What was the key moment in deciding to adopt standards?
4. Once you have made decision to join the program, how long it took to start implementing the standards?
5. Once you have started adopting quality and safety standards, how long it took to notice the first effects, if any?

**Section 6: Influence of peers in innovation decision process**

1. How important were the opinions of your peers for your decision to adopt standards?

Probe:

1. Have you waited to hear experiences of peers prior to adoption of standards?
2. Were opinions of some of your colleagues more important/influential? Why so?
3. Could you say that opinions or actions by some of your colleagues were crucial in making the decision to adopt the standards? Why?

**Section 7: Social system: attitudes of chambers and professional associations**

1. How influential were attitudes of medical chambers and professional associations for your decision to participate in certification program?

Probe:

1. Are you aware of official or unofficial position of your chamber regarding quality and safety standards?
2. Have you perceived any difference between the official and unofficial position?
3. Could you please explain, how has the chamber’s position influenced your decision to participate in certification program?
4. Are you aware of official or unofficial position of your professional associations regarding adoption of quality and safety standards?
5. Have you perceived any difference between the official and unofficial position?
6. Could you please explain, how has the professional association’s position influenced your decision to participate in certification program?

**Section 8: Conclusion**

1. Do you believe that certification program will meet its objective to improve safety and quality of health services in Republika Srpska? Please explain.

Probe:

1. What else needs to be in place in order for all the private healthcare providers to join the certification program?
2. In your opinion, who should regulate quality of private providers? How?
3. Do you believe that government should impose quality and safety standards for all health care providers? Please explain.
4. Should the certification obligation be related to Health Insurance Fund’s contract or type of practice? Should the certification process be related to ownership status of practices (such as private or public)?
5. Would you have considered joining the certification program if it had not been mandatory? Can you please explain in more details?
