# Supplementary material for: Introduction of safety and quality standards for private health care providers: a case-study from the Republic of Srpska, Bosnia and Herzegovina
Source: Int J Equity Health. 2018 Oct 5;17:92. doi: 10.1186/s12939-018-0806-0 (PMC6172732; doi:10.1186/s12939-018-0806-0)
Supplement: Supplementary file 2 — Interview guide for the private healthcare providers that have not completed certification. Interview guide used for interviews with pharmacies, specialist practices and dental practices that adopted the safety and quality standards. (DOC 74 kb) [file 12939_2018_806_MOESM2_ESM.doc]

### Interview guide for the private healthcare providers that have not completed certification

**Introduction, consent and general information**

***Note:***

Start by introducing yourself and the purpose. If this is agreeable to the interviewee, proceed to the consent form.

**General information: take notes only, not to be recorded**

1. Type of provider: 1 Pharmacy 2. Dental practice. 3 Specialist practice
2. Location municipality ………………. urban …..rural…….
3. No of employees…………
4. Contract with Health Insurance Fund: Yes …No…
5. Status (chain or independent) for pharmacies :………..
6. Years of work experience…... Years in private practice…….
7. Position in organization: 1. Owner….. 2. Managing director…...
8. Membership in professional associations …………..

Request to record interview and make sure the tape recorder is switched on. If not, detailed notes should be taken

**Section 1: Context**

1. What do you think of level of quality and safety of health care services provided in Republic of Srpska?

Probe:

1. How much are you informed about process of certification of health care institutions in Republic of Srpska?
2. What is your opinion – is there a difference between quality of services provided by private and public providers? If so can you explain why?
3. How do you perceive quality of services you provide?

**Section 2: Perceived attributes of innovation**

1. What do you think about quality and safety standards included in the certification process?

Probe:

- 1. Have you or some of your colleagues been involved in development of standards?
  2. How relevant are safety and quality standards to the health services provided by your practice/pharmacy?

1. What might be the major obstacles in implementation of quality and safety standards?
2. What benefits of certification have you observed within practices/pharmacies of your colleagues who completed the process, if any?

Probe:

1. Have you observed any drawbacks?

**Section 3: Potential consequences of not participating in the certification program**

1. Have you considered potential risks in relation with refusal to participate in certification program?

Probe:

1. Risks of harming your professional status?
2. Higher risk of harming the patients?
3. Risk of losing contract with Health Insurance Fund?
4. Risk of losing the patients?
5. Some other risks?
6. Why do you think that some of the risks are not important to you?
7. How do you protect your practice/pharmacy from these risks?

**Section 4: Communication channels and knowledge about innovation**

1. What type of information about certification was available to you during the process of deciding whether to join the certification program?

Probe:

1. What were the main sources of information (mass media, TV, radio, newspapers, professional magazines, Internet, personal contacts, seminars, professional associations, representatives of health authorities such as Agency staff or health inspectorate)? Were the information appropriate?
2. Which channel of communication you have found to be the most informative?
3. What steps have you taken to find and access relevant information? Please explain.
4. In your opinion, do you have necessary knowledge and skills to introduce the standards in your organization?

Probe:

1. Have you looked for professional assistance in order to adopt the standards?
2. Have you looked for advices from your peers?

**Section 5: innovation decision process**

1. What factors influenced you not to join the program yet?

Probe:

1. Could you estimate how long it took you to postpone joining the certification program?
2. What was the key moment in deciding to postpone joining the certification program?
3. How much has previous knowledge about certification influenced on your decision not to join the certification program yet?

**Section 6: Influence of peers in innovation decision process**

1. What role did the opinions or actions of your peers play in your decision not to participate in the program yet?

Probe:

1. Have you waited to hear experiences of your peers prior to making your decision?
2. How important were opinions of your peers, in comparison to other sources of information?
3. Were opinions of some of your colleagues more important/influential? Please explain why.

**Section 7: Social system: attitudes of chambers and professional associations**

1. How influential were attitudes of medical chambers and professional associations for your decision to postpone participation in certification program?

Probe:

1. Are you aware of official or unofficial position of your chamber regarding quality and safety standards?
2. Have you perceived any difference between the official and unofficial position?
3. Could you please explain, how has the chamber’s position influenced your decision on postponing participation in certification program?
4. Are you aware of official or unofficial position of your professional associations regarding adoption of quality and safety standards?
5. Have you perceived any difference between the official and unofficial position?
6. Could you please explain, how has the professional association’s position influenced your decision to postpone participation in certification program?

**Section 8: Conclusion**

1. Do you believe that certification program will meet its objective to improve safety and quality of health services in Republika Srpska? Please explain.
2. Who should regulate quality of private providers? How?
3. Do you believe that government should impose quality and safety standards for all health care providers? Please explain.
4. Should the certification obligation be related to Health Insurance Fund’s contract or type of practice? Should the certification process be related to ownership status of practices (such as private or public)?
5. Would you have considered joining the certification program if it had not been mandatory? Can you please explain in more details?
6. What else needs to be in place in order for you to reconsider join the certification program?
