# Supplementary material for: Introduction of safety and quality standards for private health care providers: a case-study from the Republic of Srpska, Bosnia and Herzegovina
Source: Int J Equity Health. 2018 Oct 5;17:92. doi: 10.1186/s12939-018-0806-0 (PMC6172732; doi:10.1186/s12939-018-0806-0)
Supplement: Supplementary file 3 — Questionnaire for the private healthcare providers. The questionnaire used in self-administered postal survey of pharmacies, specialist practices and dental practices. (DOC 190 kb) [file 12939_2018_806_MOESM3_ESM.doc]

**QUESTIONNAIRE FOR THE PRIVATE HEALTHCARE PROVIDERS**

**PLEASE PROVIDE THE FOLLOWING GENERAL INFORMATION:**

**Type of provider:** 1 🞏 Pharmacy 2 🞏 Specialist practice 3 🞏 Dental practice

**Tick status of your organization (for pharmacies only):**

1 🞏 Chain of pharmacies 2 🞏 Independent pharmacy

**Position in organization:** 1 🞏 Owner 2 🞏 Managing director 3 🞏 Owner & Managing director

🞏 Other: ______________

**When was your practice/pharmacy established:** _____ year

**Number of employees in your practice/pharmacy:** ________

**Does your practice/pharmacy have contract with Health Insurance Fund:**

1 🞏 Yes 2 🞏 No

**Has your practice/pharmacy completed certification process:**
 1 🞏 Yes 2 🞏 No 3 🞏 The certification is ongoing

If you completed the certification process, provide the month and year of completion:

_____________________________________________________________________

**Membership in professional associations (tick all that apply):**

1 🞏 Association of medical doctors in private practice of the Republic of Srpska

2 🞏 Pharmaceutical society of the Republic of Srpska

3 🞏 Association of private practice dentist of the Republic of Srpska

4 🞏 Association of specialized dentist of the Republic of Srpska

5 🞏 None

**IN THE FOLLOWING PART OF THE QUESTIONNARE WE PRESENTED CLAIMS THROUGH WHICH WE EXSAMINES YOUR ATTITUDE AND EXPERIENCE RELATED TO THE CERTIFICATION PROCESS. PLEASE CHECK BOXES TO CHOOSE ONE OF AVAILABLE RESPONSES IN RELATION TO STATEMENTS:**

|  | | **Strongly disagree** | **Partially disagree** | **Neither agree nor disagree** | **Partially agree** | **Strongly agree** |
| --- | --- | --- | --- | --- | --- | --- |
| **Relative advantages and disadvantages of certification process** | | | | | | |
|  | Certification facilitates and improves system of work. | 🞏 | 🞏 | 🞏 | 🞏 | 🞏 |
|  | Certification process is a financial burden for the organization. | 🞏 | 🞏 | 🞏 | 🞏 | 🞏 |
|  | Certification process improves safety and quality of healthcare providers’ services. | 🞏 | 🞏 | 🞏 | 🞏 | 🞏 |
|  | Certification adds a lot of extra administration work. | 🞏 | 🞏 | 🞏 | 🞏 | 🞏 |
|  | Certification process takes time from provision of services to patients. | 🞏 | 🞏 | 🞏 | 🞏 | 🞏 |
|  | Certification process facilitates job orientation. | 🞏 | 🞏 | 🞏 | 🞏 | 🞏 |
|  | Certification facilitates management of the healthcare provider organizations. | 🞏 | 🞏 | 🞏 | 🞏 | 🞏 |
| **Observability of the certification process** | | | | | | |
|  | Patients observe the differences in functioning of certified health care providers. | 🞏 | 🞏 | 🞏 | 🞏 | 🞏 |
|  | Health Insurance Fund positively values certified healthcare providers. | 🞏 | 🞏 | 🞏 | 🞏 | 🞏 |
|  | Health Inspection positively values certified healthcare providers. | 🞏 | 🞏 | 🞏 | 🞏 | 🞏 |
|  | Ministry of Health and Social Welfare positively values certified healthcare providers. | 🞏 | 🞏 | 🞏 | 🞏 | 🞏 |
|  | Certified healthcare providers are recognized in the public as an example of good practice. | 🞏 | 🞏 | 🞏 | 🞏 | 🞏 |
| **Certification standards characteristics** | | | | | | |
|  | Certification standards can be implemented in my practice/pharmacy. | 🞏 | 🞏 | 🞏 | 🞏 | 🞏 |
|  | Certification standards should be better tailored to the type and size of practice/pharmacy. | 🞏 | 🞏 | 🞏 | 🞏 | 🞏 |
|  | Requirements of the certification standards are clearly defined. | 🞏 | 🞏 | 🞏 | 🞏 | 🞏 |
|  | Certification standards are too voluminous and broad. | 🞏 | 🞏 | 🞏 | 🞏 | 🞏 |
|  | Certification standards are relevant to the services provided by my practice/pharmacy. | 🞏 | 🞏 | 🞏 | 🞏 | 🞏 |
| **Knowledge: Access to information on certification process** | | | | | | |
|  | Adequate information about certification was available to me at the time of deciding whether to join the certification process. | 🞏 | 🞏 | 🞏 | 🞏 | 🞏 |
|  | We asked the different healthcare system institutions about everything unclear in relation to the certification process. | 🞏 | 🞏 | 🞏 | 🞏 | 🞏 |
|  | All information about certification process was available at the ASKVA’s web site. | 🞏 | 🞏 | 🞏 | 🞏 | 🞏 |
|  | ASKVA sufficiently informed healthcare providers about the certification process | 🞏 | 🞏 | 🞏 | 🞏 | 🞏 |
|  | Healthcare providers were mostly left for themselves to seek information about the certification process. | 🞏 | 🞏 | 🞏 | 🞏 | 🞏 |
| **Social system: Professional associations influence** | | | | | | |
|  | Professional association had positive attitude towards the certification process. | 🞏 | 🞏 | 🞏 | 🞏 | 🞏 |
|  | Professional association was interested in the certification process | 🞏 | 🞏 | 🞏 | 🞏 | 🞏 |
|  | Professional association supported me in preparation for the certification. | 🞏 | 🞏 | 🞏 | 🞏 | 🞏 |
|  | Professional associate clearly expressed its position on the certification process. | 🞏 | 🞏 | 🞏 | 🞏 | 🞏 |
|  | Professional association provided all relevant information about the certification process | 🞏 | 🞏 | 🞏 | 🞏 | 🞏 |
| **Interpersonal communication channels** | | | | | | |
|  | My peers had clear attitudes towards the certification process | 🞏 | 🞏 | 🞏 | 🞏 | 🞏 |
|  | My peers had had positive attitude towards the certification process | 🞏 | 🞏 | 🞏 | 🞏 | 🞏 |
|  | My peers were interested in the certification. | 🞏 | 🞏 | 🞏 | 🞏 | 🞏 |
|  | My attitude towards certification was mostly formed in contacts with peers who completed the process | 🞏 | 🞏 | 🞏 | 🞏 | 🞏 |
| **Social system: Professional chambers influence** | | | | | | |
|  | Medical chamber had positive attitude towards the certification process. | 🞏 | 🞏 | 🞏 | 🞏 | 🞏 |
|  | Medical chamber was interested in the certification process | 🞏 | 🞏 | 🞏 | 🞏 | 🞏 |
|  | Medical chamber supported me in preparation for the certification. | 🞏 | 🞏 | 🞏 | 🞏 | 🞏 |
|  | Medical chamber clearly expressed its position on the certification process. | 🞏 | 🞏 | 🞏 | 🞏 | 🞏 |
|  | Medical chamber provided all relevant information about the certification process | 🞏 | 🞏 | 🞏 | 🞏 | 🞏 |
| **Persuasion: The willingness for accepting certification process** | | | | | | |
|  | We would join the certification program even if it had not been mandatory. | 🞏 | 🞏 | 🞏 | 🞏 | 🞏 |
|  | We would recommend the certification to all healthcare providers. | 🞏 | 🞏 | 🞏 | 🞏 | 🞏 |
|  | We prefer different work methods in our organisations rather than one that is offered by the certification process. | 🞏 | 🞏 | 🞏 | 🞏 | 🞏 |
|  | I would recommend introduction of the certification standards to my peers. | 🞏 | 🞏 | 🞏 | 🞏 | 🞏 |
|  |  | (1) | (2) | (3) | (4) | (5) |

**PLEASE PROVIDE YES OR NO ANSWERS TO THE QUESTIONS 41-49:**

1. **Have you expected (or do you expect) any gains from the certification**?

| 1) | | Gains in professional status | Yes | 🞏 | No | 🞏 | |
| --- | --- | --- | --- | --- | --- | --- | --- |
| 2) | | Gains related to patient’s satisfaction | Yes | 🞏 | No | 🞏 | |
| 3) | | Gains related to staff satisfaction | Yes | 🞏 | No | 🞏 | |
| 4) | | Gaining additional patients | Yes | 🞏 | No | 🞏 | |
| 5) | | Advantages in contracting with the Health Insurance Fund of the Republic of Srpska | Yes | 🞏 | No | 🞏 | |
| 6) | | Other gains:_________________________ | Yes | 🞏 | No | 🞏 | |
|  | |  |  | (1) |  | (2) | |
|  | If you have expected/expect some **gains** which were the most important to you: _______________________________________________________________________ | | | | | |  |

1. **Did you achieve any benefits from certification process (TO BE COMPLETED ONLY BY CERTIFIED PRIVATE PRACTICES**)?

| 1) | Benefit in the professional status | Yes | 🞏 | No | 🞏 |
| --- | --- | --- | --- | --- | --- |
| 2) | Benefits related to patient’s satisfaction | Yes | 🞏 | No | 🞏 |
| 3) | The benefits related to related to staff satisfaction | Yes | 🞏 | No | 🞏 |
| 4) | The benefits from attracting additional patients | Yes | 🞏 | No | 🞏 |
| 5) | Advantages in contracting with the Health Insurance Fund of the Republic of Srpska | Yes | 🞏 | No | 🞏 |
| 6) | Other benefits:_________________________ | Yes | 🞏 | No | 🞏 |
|  |  |  | (1) |  | (2) |

If you have expected/expect some benefits which were the most important to you: ________________________________________________________________________

____________________________________________________________________________

1. **Which of the risks did/can the certification process mitigate in your practice/pharmacy?**

Which of the risks was/is the most important to you: ____________________________________

| 1) | Risk of harming the patients | Yes | 🞏 | No | 🞏 |
| --- | --- | --- | --- | --- | --- |
| 2) | Risk of losing contract with Health Insurance Fund | Yes | 🞏 | No | 🞏 |
| 3) | Risk of losing patients | Yes | 🞏 | No | 🞏 |
| 4) | Risk of paying fines resulting from Inspectorate’s visit | Yes | 🞏 | No | 🞏 |
| 5) | Risk of having court processes initiated by patients | Yes | 🞏 | No | 🞏 |
| 6) | Risk of staff professional diseases and injuries | Yes | 🞏 | No | 🞏 |
| 7) | Other risks: _______________________________ | Yes | 🞏 | No | 🞏 |
|  |  |  | (1) |  | (2) |

______________________________________________________________________________

1. **How did you obtain the information about the certification process?**

| 1) | Through TV, radio and newspaper | Yes | 🞏 | No | 🞏 |
| --- | --- | --- | --- | --- | --- |
| 2) | Through professional magazines | Yes | 🞏 | No | 🞏 |
| 3) | Through Official Gazette | Yes | 🞏 | No | 🞏 |
| 4) | Through Internet | Yes | 🞏 | No | 🞏 |
| 5) | Through direct contact with my peers | Yes | 🞏 | No | 🞏 |
| 6) | Through contacts with certified healthcare providers | Yes | 🞏 | No | 🞏 |
| 6) | Through professional associations | Yes | 🞏 | No | 🞏 |
| 7) | Through chamber | Yes | 🞏 | No | 🞏 |
| 8) | Through professional meetings and seminars | Yes | 🞏 | No | 🞏 |
| 9) | Through contacts with representatives of ASKVA | Yes | 🞏 | No | 🞏 |
| 10) | Through contacts with representatives Public Health Institute | Yes | 🞏 | No | 🞏 |
| 11) | Through contacts with representatives Ministry of Health and Social Welfare | Yes | 🞏 | No | 🞏 |
| 12) | Through other sources: ______________________ | Yes | 🞏 | No | 🞏 |
|  |  |  | (1) |  | (2) |

Which of the sources was/is the most important to you:_____________________________

__________________________________________________________________________

|  | **Did the medical chamber influence your decision whether to accept the certification process?** | Yes | 🞏 | No | 🞏 |
| --- | --- | --- | --- | --- | --- |
|  | **Did the professional association influence your decision whether to accept the certification process?** | Yes | 🞏 | No | 🞏 |
|  | **Did you ask for advice from your peers in relation to the certification standards?** | Yes | 🞏 | No | 🞏 |
|  | **Have you waited to hear experiences your peers before deciding whether to join to the certification process?** | Yes | 🞏 | No | 🞏 |
|  | **Have the opinions and actions of your peers had influence on your decision to accept the certification process?** | Yes | 🞏 | No | 🞏 |
|  |  |  | (1) |  | (2) |

| Space for additional observations or comment you’d like share with us, including those related to the content and structure of questions in the questionnaire. |
| --- |
